# Cone beam CT of the musculoskeletal system: clinical applications

**DOI:** 10.1007/s13244-017-0582-1

**Published:** 2018-01-04

**Authors:** Magdalena Posadzy, Julie Desimpel, Filip Vanhoenacker

**Affiliations:** 1 Department of Radiology, W. Dega Orthopaedic and Rehabilitation University Hospital of Karol Marcinkowski University of Medical Sciences, Poznan, Poland; 2 Department of Radiology, Antwerp University Hospital, Antwerp University, Antwerp, Belgium; 3 Department of Radiology, AZ Sint-Maarten, Mechelen, Belgium; 4 Faculty of Medicine and Health sciences, Ghent University, Ghent, Belgium

**Keywords:** Cone beam computed tomography, Multidetector computed tomography, Conventional radiography, Magnetic resonance imaging, Musculoskeletal imaging

## Abstract

**Objectives:**

The aim of this pictorial review is to illustrate the use of CBCT in a broad spectrum of musculoskeletal disorders and to compare its diagnostic merit with other imaging modalities, such as conventional radiography (CR), Multidetector Computed Tomography (MDCT) and Magnetic Resonance Imaging.

**Background:**

Cone Beam Computed Tomography (CBCT) has been widely used for dental imaging for over two decades.

**Discussion:**

Current CBCT equipment allows use for imaging of various musculoskeletal applications. Because of its low cost and relatively low irradiation, CBCT may have an emergent role in making a more precise diagnosis, assessment of local extent and follow-up of fractures and dislocations of small bones and joints. Due to its exquisite high spatial resolution, CBCT in combination with arthrography may be the preferred technique for detection and local staging of cartilage lesions in small joints. Evaluation of degenerative joint disorders may be facilitated by CBCT compared to CR, particularly in those anatomical areas in which there is much superposition of adjacent bony structures. The use of CBCT in evaluation of osteomyelitis is restricted to detection of sequestrum formation in chronic osteomyelitis. Miscellaneous applications include assessment of (symptomatic) variants, detection and characterization of tumour and tumour-like conditions of bone.

***Teaching Points*:**

*• Review the spectrum of MSK disorders in which CBCT may be complementary to other imaging techniques.*

*• Compare the advantages and drawbacks of CBCT compared to other imaging techniques.*

*• Define the present and future role of CBCT in musculoskeletal imaging.*

## Introduction

**Fig. 1:**
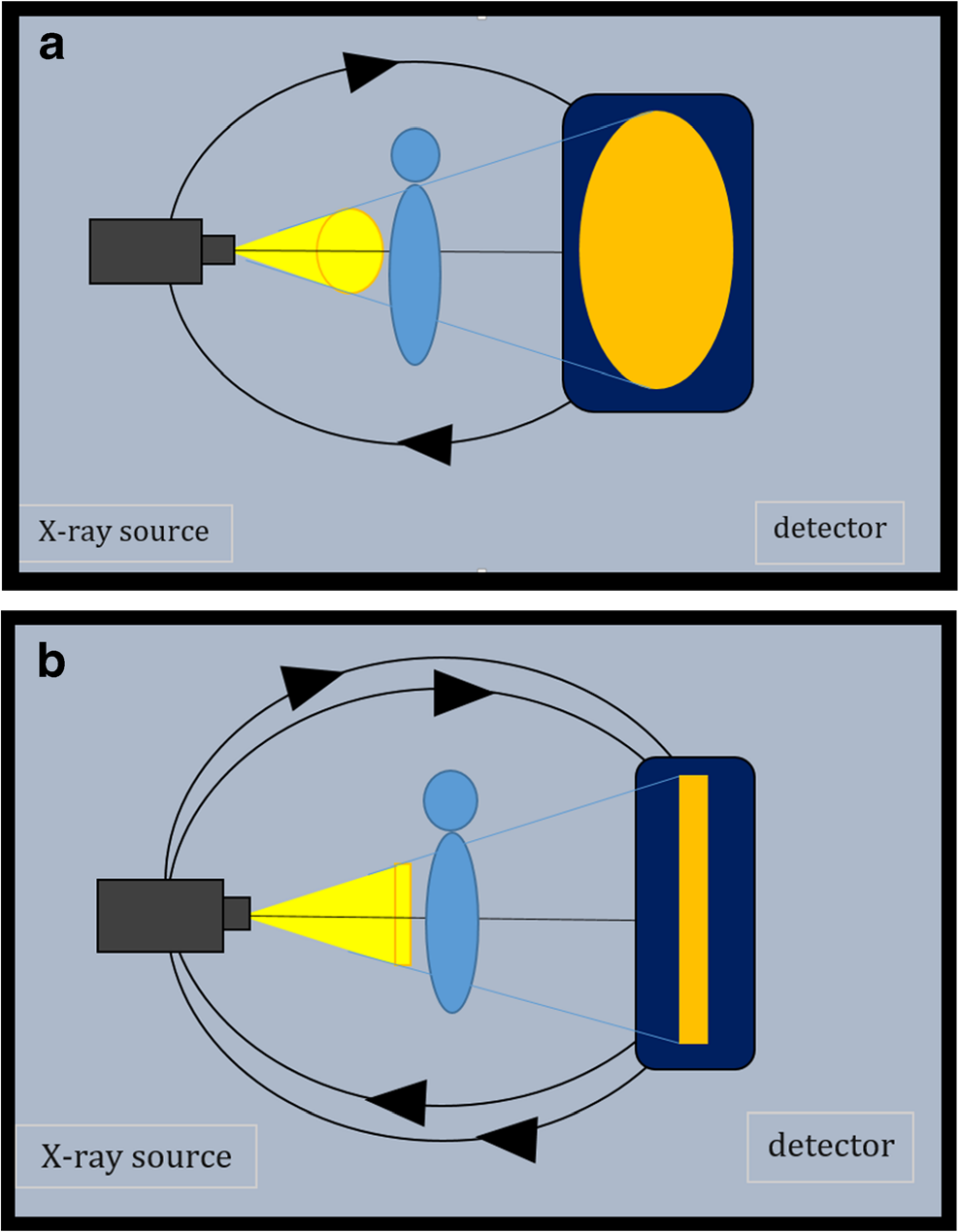
Principle of CBCT and MDCT. **a** In CBCT, cone-shaped X-ray beam reaches a flat detector after a single rotation of the gantry around the patient. **b** In MDCT, narrowly collimated, fan-shaped beam and multiple linear detectors rotate around the patient to acquire multiple image sections per rotation. In both techniques volumetric images are reconstructed into a 3-D volume dataset of images

Although initially used for dental imaging, Cone Beam Computed Tomography (CBCT) is currently installed in many radiology departments as an integral part of the imaging armamentarium. CBCT uses a conical x-ray beam which falls on a flat panel detector unlike conventional Multidetector Computed Tomography (MDCT), where a fan shaped beam and linear detectors are used (Fig. 1). In CBCT, the X-ray tube and the detector synchronously rotate 360° around the patient. At certain degree intervals, single projection images or “basis” images, are acquired. Software programs incorporating sophisticated algorithms including back-filtered projection are applied to these projection data to generate a volumetric data set, which can be used for reconstruction images in three orthogonal planes [1]. In our department, we use a CBCT with a gantry of 58 cm patient aperture and a movable table allowing horizontal positioning and multifunctional use (NewTom 5 G, QR systems, Verona, Italy). The specific purpose of this paper is to present a pictorial overview of the clinical usefulness of the CBCT of evaluation in a broad spectrum of musculoskeletal disorders and to compare its diagnostic merit with other imaging modalities, such as conventional radiography (CR), Multidetector Computed Tomography (MDCT) and Magnetic Resonance Imaging (MRI).

## Advantages and disadvantages

**Fig. 2:**
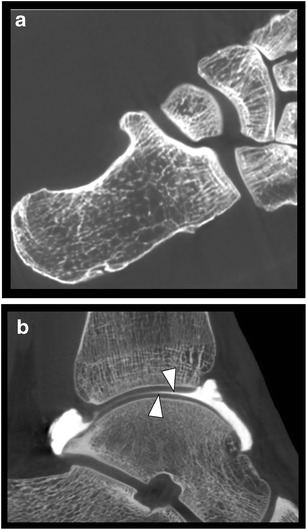
Evaluation of bone architecture and normal articular cartilage. **a**. Axial CBCT image of a cadaver foot illustrating exquisite detail of the cortical and trabecular bone architecture. **b**. Sagittal reformatted image of a CBCT-A of the talocrural joint showing smooth surface of normal articular cartilage surface of distal tibia and talar dome (arrowheads)

A major advantage of CBCT is its high spatial resolution resulting in exquisite detail of bone microarchitecture (Fig. 2a) [2, 3]. CBCT after intra-articular contrast injection (CBCT arthrography; CBCT-A) offers high resolution images of the articular cartilage surface. (Fig. 2b) [4]. With our equipment, the spatial resolution ranges between 300 μm for a standard scan to 75 μm for high resolution images. Recent studies, performed on phantoms and also in a cohort of paediatric patients, confirm the significant lower dose of CBCT, compared to MDCT [5–11]. Effective dose for paranasal sinuses imaging in CBCT is approximately 40% lower than standard MDCT and 30% lower than low-dose sinus CT scans [12]. Studies on phantoms in the ankle region showed 21.4 μSv of effective dose for MDCT, for CBCT it was reported ranging from 1.9 μSv to14.3 μSv [11]. Lower radiation dose results from single rotation of the gantry required for acquisition of the whole scan volume, smaller field of view, pulsed X-ray beams instead of the constant radiation stream and the use of a large high quality flat panel detector [13].

Our NewTom 5 G equipment uses a fixed tube voltage of 110 kVp, but is fitted with the SafeBeam technology™, allowing reduction of the radiation dose.

Initial optimization of the tube current (mA) occurs on estimation of the patient size based on the attenuation information derived from an anteroposterior and lateral scout views [11].

Angular tube current modulation further equalizes in real time the photon flux to the detector as the X-ray tube rotates about the patient among the anteroposterior and lateral position according to the measured attenuation from the previous projection, allowing further adaptation of the dose for the anatomy of the patient [14, 15].

By using the pulsed emission technology, exposure is restricted to intermittent bursts of radiation for each degree instead of using a constant stream of radiation during the 360 ° rotation. This results in a considerable decrease of effective exposure time (e.g., if a 360 ° rotation lasts for 18 to 36 s, the effective exposure time during the 360° rotation is 2.4 to 7.3 s) [16]. This does not affect the overall image quality as the dataset obtained from 360 projections (one for each degree during 360° rotation) may be used for qualitative image reconstruction.

For small joints, the spectrum of the effective dose (ED) in CBCT ranges between 1 to 15.3 μSv applying a conversion factor of 0.01 mSv/Gy x cm^2^ for peripheral joints, which is significantly lower than values reported for MDCT. Among those imaging methods using radiation, Conventional Radiography (CR) still remains the one with the lowest ED, between 0.07 to 5 μSv) [17].

However, image quality of CT and CBCT images may be altered by implanted metal elements reducing the contrast, obscuring structures and impairing the detection of areas of interest. This image degradation may be reduced by using dedicated algorithms and software [18, 19]. The overall cost of the equipment is far less than MDCT [20], thus it is suited for private practices or small medical centres or as additional CT equipment in large institutions. Table 1 provides a short overview of the parameters that may influence the economic rentability of installing a CBCT. However, as the referral pattern for certain examinations may differ among different health care centres and reimbursement can significantly differ depending on the health policy of each country, it is not possible to provide general recommendations.

**Table 1:** Comparison of parameters influencing the economic rentabilty of CBCT versus MDCT

	CBCT (high-end)	MDCT (mid-end)
Purchase price equipment*	21–30% (200,000 Euro)	(665,000–968,000 Euro)
Annual maintenance service*	10% (10,000 Euro)	(100,000 Euro)
Required area for placement of equipment + operating space for medical staff (minimum versus our hospital)	16.5 m^2^–33 m^2^	36 m^2^–52 m^2^
Investment cost for room preparation	100,000 Euro	150,000 Euro
Operating cost (electricity, other utilities)	50%	100%
Cost medical staff	Similar (10 min acquisition and reconstruction time)	Similar (10 min acquisition and reconstruction time)
Honorarium examination	Similar (e.g. 50.90 Euro for MSK examination)**	Similar (e.g. 50.90 Euro for MSK)
Other applications than small bones/joints	Dental, petrous bone, sinuses	Brain, Spine, Abdomen, Chest, Bone and Joints (including large joints), CT-angiography, …

*Based on list price provided by the manufacturer of our high-end CBCT equipment versus a MDCT mid-end equipment (range of different manufacturers)

**Currently pending approval of the National Institute for Sickness and Invalidity Insurance of our country

Abbreviations: m = meter; m^2^ = square meter; MSK = musculoskeletal; % = percentage

A disadvantage of CBCT is the limited field of view (FOV), which ranges from 6 × 6 cm to maximum 18 × 16 cm with our equipment. Therefore, CBCT is not suitable for imaging of large joints. Another drawback is its limitation to assess soft tissue pathology due to lack of contrast resolution and Hounsfield Units (HU) measurements. Furthermore, CBCT is more time consuming (18 to 36 s of acquisition time) resulting in higher susceptibility to motion artifacts. Table 2 summarizes the main advantages and disadvantages of musculoskeletal CBCT.

**Table 2:** Advantages and disadvantages of musculoskeletal CBCT

ADVANTAGES	DISADVANTAGES
Lower radiation dose than MDCT	Radiation exposure higher than CR
More comfortable positioning than MRI for patients suffering from claustrophobia	Prone to motion artifacts (patient with tremor, children)
Suitable for postoperative follow-up in patient with metallic implants using appropriate metal artifact algorithms	Limited field of view
High spatial resolution images of bone architecture	Limited evaluation of soft tissue pathology
High spatial resolution images of cartilage surface after intra-articular contrast injection	Mildly more time consuming than CR, comparable examination time to MDCT due to easier positioning
Relative low cost of equipment	
Joint imaging in weight-bearing position with some CBCT equipment	

## Evaluation of fractures, dislocations and their follow-up

**Fig. 3:**
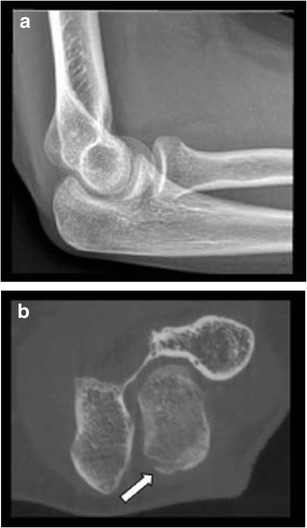
Occult olecranon fracture. **a**. Lateral view CR of the elbow shows no evidence of fracture. **b**. Axial CBCT reformatted image reveals subtle cortical disruption and a small adjacent bone fragment at the posterior aspect of the olecranon process

**Fig. 4:**
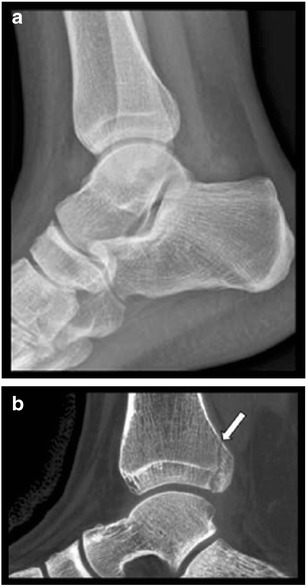
Fracture of the posterior malleolus in a 16-year-old female. **a**. CR (lateral view) of the ankle shows no evidence of fracture. **b**. Sagittal CBCT reconstruction showing a non-displaced malleolus tertius fracture (arrow). CBCT clearly demonstrates intra-articular extension of the fracture

**Fig. 5:**
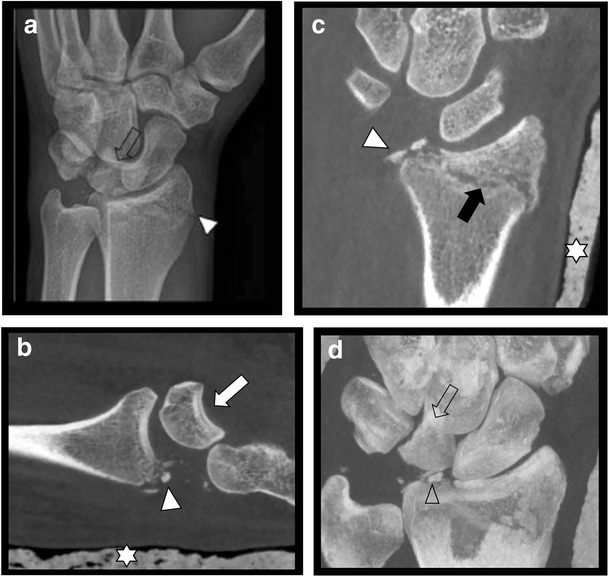
Complex fracture of the dorsal side of the styloid process of the radius with intra-articular involvement, multiple intra-articular fracture fragments and perilunate dislocation. **a**. CR (oblique view) shows a fracture of the distal radius (arrowhead) and perilunate dislocation (arrow). **b**. Sagittal CBCT reformatted image after immobilization and casting (stars) shows residual perilunate dislocation (white arrow) with dorsal displacement of the distal carpal row and additional fracture fragments (arrowhead). **c**. Coronal CBCT reformatted image after immobilization and casting (stars) demonstrates distal radius fracture (black arrow). The degree of communition and additional fracture fragments better seen than on plain films (arrowhead). **d**. 3-D reconstruction may be useful for evaluation of the displacement of the carpal bones (open arrow) and additional fracture fragments (open arrowhead)

CR is the first-line imaging technique in case of clinical suspicion of fractures, although being less sensitive than cross-sectional imaging. CBCT imaging shows higher sensitivity in detection of small bone and joint trauma than CR and may visualize fractures being occult on CR (Fig. 3 and Fig. 4) or confirm doubtful fractures [17, 21, 22]. In most cases, this has an impact on the treatment strategy [5]. Comparison of MDCT as the gold standard with CBCT for finger fractures showed similar results in depicting the fracture and assessment of articular involvement [7]. In case of high clinical suspicion of carpal fractures (especially the scaphoid bone), but negative CR, subsequent MRI is often recommended for exclusion of occult nondisplaced fractures and bone marrow contusion. In this scenario, MRI remains more sensitive than CBCT [21]. Nonetheless, it is not always possible to perform MRI immediately following trauma and MRI cannot be performed in every patient, due to potential contraindications or lesser accessibility. Therefore, CBCT should be considered as a second line imaging modality in assessing complex anatomical sites with multiple overlapping bones, such as the wrist (Fig. 5) and foot, in case of negative CR but with high clinical suspicion of a fracture. Prompt, accurate evaluation of fractures may obviate the need of MRI at a later date [21] .

In case of suspicion of joint instability, weight-bearing CBCT provides information about joint alignment [5, 8] although this can only be performed on dedicated CBCT equipment [20].

For follow-up of bone healing and callus formation, CR may be difficult, especially in the presence of overlying splints or casts. As CBCT can provide more detailed information on bone architecture in comparison to CR, it can also help in evaluation of the healing process, which can be over- or underestimated on CR [6]. As a cross-sectional technique and the possibility for multidirectional reformations and 3-D reconstructions, CBCT is superior to CR in assessment of callus formation, osseous bridging and evaluation of residual fracture lines. In case of postoperative follow-up after placement of metallic hardware, incomplete healing as well as early detection of hardware loosening may be facilitated by CBCT. For hardware fractures, however, CR still remains the preferred method because of potential metallic streak artifacts on CBCT. The possible explanation for this potential discrepancy is related to the size of the metal objects. Indeed, at the bone–screw interface, higher contrast and spatial resolution of CBCT dominates the effect of the beam hardening owing to the relatively small size of metallic screws. Conversely, the beam hardening artifact surrounding large side plates in CBCT images compared with plain radiograph dominates the effect of better contrast resolution on CBCT [23]. Despite the use of current Metal Artifact Reduction sequences, the overall usefulness of MRI after screw fixation is limited due to metal artifacts.

## Bone tumours and tumour-like lesions

**Fig. 6:**
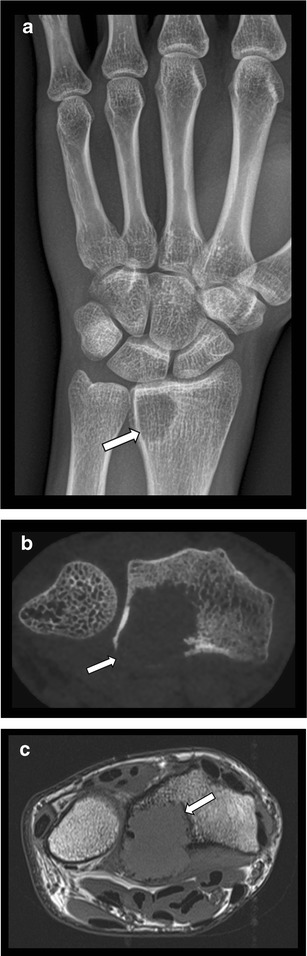
Giant cell tumour of distal radius. **a**. CR (AP view) showing an osteolytic lesion in the distal epiphysis of the radius (arrow). There is no major cortical breakthrough visible on CR. **b**. Axial CBCT reconstruction clearly show cortical breakthrough of the lesion. The precise extent of the soft tissue involvement is inaccurate due to insufficient soft tissue contrast. **c**. On axial T1-WI MRI, the lesion is isointense to muscle with cortical disruption at the volar aspect and involvement of pronator quadratus muscle. MRI is far superior for evaluation of the soft tissue component of the lesion

The value of CBCT in the assessment of tumour and tumour-like conditions of the jaw bones has been reported previously in the dental literature [24–26]. Compared to the limited 2-dimensional information of conventional panoramic view, CBCT provides more precise information on location, morphology, intra-osseous extent, cortical breakthrough periosteal reaction and local effect on adjacent structures and teeth roots. Although definitive characterization of these lesions is often difficult or even impossible due to overlapping imaging characteristics, analysis of the matrix and intralesional calcifications and relationship with dentition are very helpful parameters in identification of odontogenic and non- odontogenic tumour and tumour-like conditions of the jaws. A disadvantage of CBCT is its limited assessment of the potential soft tissue component of the lesions. For bone tumours located in extremities, the use of CBCT is less documented. Similar to lesions in the jaws, CBCT allows for a more accurate evaluation of lesion location (either in the longitudinal or transverse axis of the bone), cortical breakthrough and periosteal reaction than CR (Fig. 6). Although these semiological features may help in lesion characterisation, MDCT and especially MRI are better suited for evaluation of the soft tissue component. Therefore, CBCT cannot be regarded as preferred technique for assessment of bone tumours.

## Osteomyelitis

**Fig. 7:**
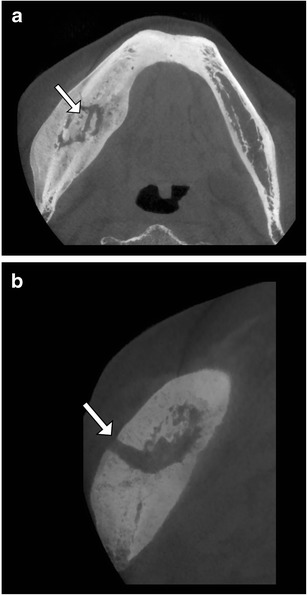
Chronic osteomyelitis of the right hemimandible. **a**. Axial CBCT reformatted image showing chronic osteomyelitis of the right hemimandible with intralesional sequestrum (arrow). Note marked sclerosis of the right hemimandible compared to the left side and massive periosteal bone reaction (involucrum). **b**. Detailed axial view shows the course of a fistula through the mandibular cortex to the buccal soft tissues (arrow)

Although the preferred imaging modalities in evaluation of osteomyelitis are CR as baseline examination for follow-up and MRI for early detection and staging, CT is the best technique for assessment of a sequestrum in chronic osteomyelitis (Fig.7). In the jaw bones and appendicular skeleton, CBCT may have an equal diagnostic performance as MDCT. On MRI, a sequestrum is difficult to distinguish from sclerotic but viable bone. CBCT can also provide more detailed visualization of osteolytic changes caused by infection in the presence of metallic hardware.

## Degenerative joint disease

**Fig. 8:**
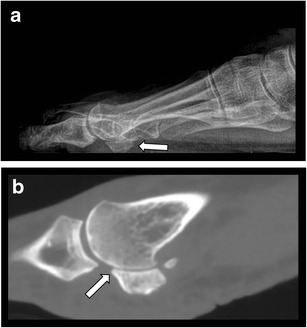
Sesamoid degenerative changes. **a**. CR lateral view of the foot in standing position with suspected degenerative changes between the sesamoids and the first metatarsal head. **b**. CBCT sagittal reformatted image shows osteophyte formation, narrowing of the joint space and subchondral sclerosis at the joint between the plantar aspect of the metatarsal head and the medial sesamoid (arrow)

**Fig. 9:**
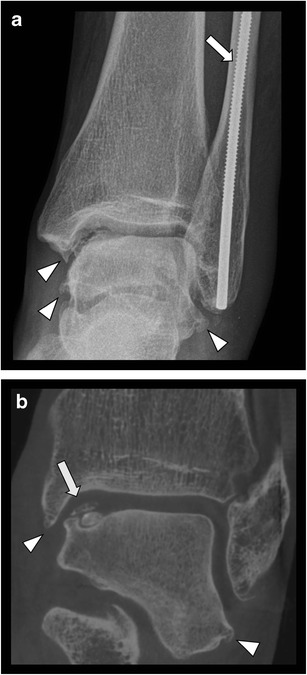
Posttraumatic unstable osteochondral lesion of the talus and massive degenerative changes of the ankle in a 66-year-old female. **a**. CR (AP view) demonstrating the presence of metallic screw within the fibula (arrow). Note irregular articular surface of talar dome and advanced degenerative changes with osteophytes formation (arrowheads). **b**. CBCT coronal reformatted image better shows the presence and extent of an unstable osteochondral lesion (arrow) and osteophytes (arrowheads). There is no metal artifact from the screw in the fibula

In comparison to CR, CBCT shows more precisely the degree and extent of degenerative joint changes. It can depict pathology in small joints not visible due to overlying of bony structures, for example, in sesamoid bones at the level of metatarsal head (Fig.8) or in the presence of a metallic screw (Fig.9). Small osteophytes, joint space narrowing and subtle areas of subchondral sclerosis can be detected. Evaluation of this subtle cartilage loss in small joints on MRI is far more challenging because of poor spatial resolution. However, bone marrow oedema indicating disease activity is only detected on MRI.

## CBCT arthrography

**Fig. 10:**
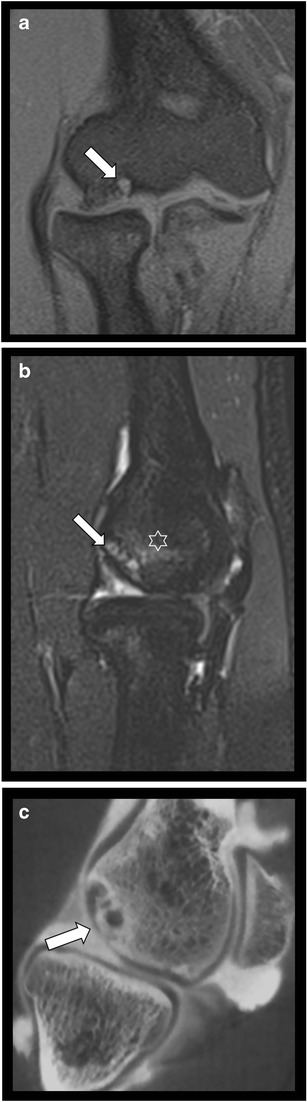
Osteochondral lesion of the capitulum. **a**. Coronal T1-WI fat saturated MR image showing a osteochondral lesion of the capitulum. **b**. Sagittal PD MRI fat saturated image at the level of radio-humeral joint revealing subchondral bone marrow changes (star) and subchondral cyst formation (arrow), the cartilage cannot be evaluated precisely. **c**. Sagittal reformatted CBCT-A at the level of the radio-humeral joint showing the osteochondral lesion of the capitulum with subchondral cyst formation, surrounding sclerosis and subtle focal thinning of the articular cartilage (arrow)

**Fig. 11:**
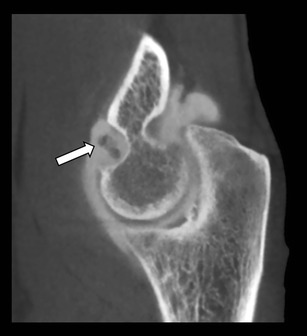
Intra-articular loose bodies. Sagittal CBCT-A reformatted image shows the presence of intra-articular loose bodies (arrow)

**Fig. 12:**
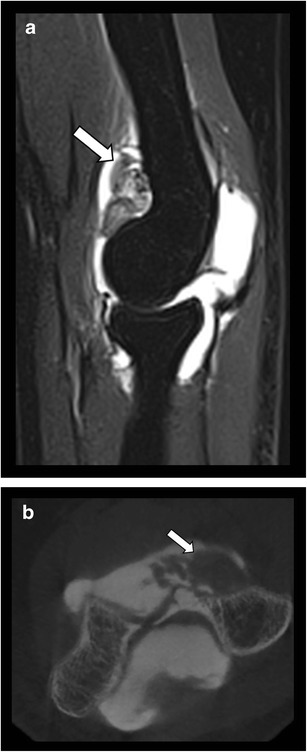
Pigmented villonodular synovitis of the elbow. **a**. Sagittal T2–WI fat suppressed MRI image presenting a mass within the joint cavity (arrow) with a low signal areas related to hemosiderin deposits. **b**. CBCT-A showing the presence of proliferative synovium (arrow) within the anterior part of the joint cavity

To evaluate chondral lesions, arthroscopy is a reference standard procedure offering also simultaneous treatment. Considering the operative risk and invasive nature of arthroscopy, appropriate preoperative imaging is preferable for diagnosis and local staging of cartilage lesions. For some cartilage lesions MR-arthrography (MRA) is widely used, particularly at the wrist. CBCT arthrography has -however- a better spatial resolution, the ability for thin multiplanar reformats allowing more accurate staging of articular cartilage lesions (Fig.10). This method can be notably useful in case of orthopaedic implants located near the area of interest [9, 27], in which MRI is less feasible due to susceptibility artifacts [27]. Intra-articular loose bodies (Fig. 11) or synovial tumour and tumour-like conditions such as Pigmented Villonodular Synovitis (PVNS) can be also better visualized after intra-articular contrast injection (Fig. 12).

## Miscellaneous

**Fig. 13:**
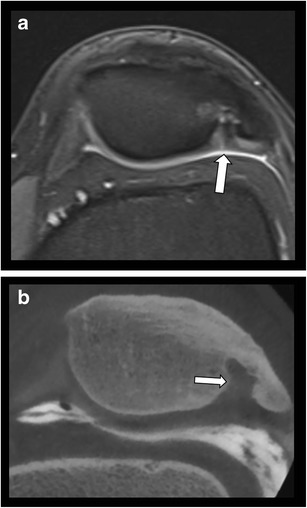
Dorsal defect of the patella simulating a large cartilage defect on MRI. **a**. Axial T2 WI fat saturated MR image shows a focal bony defect with surrounding bone marrow edema at the superolateral aspect of the patella. There is suspicion of an overlying cartilage fissure (arrow). **b**. Axial reformatted CBCT-A demonstrates a dorsal patella defect. The overlying patellar cartilage is intact.

**Fig. 14:**
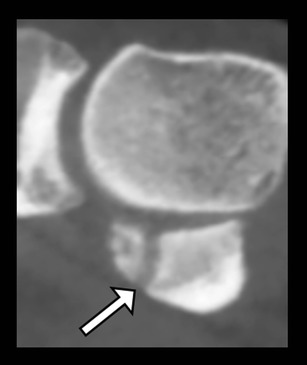
Bifid medial sesamoid bone of the hallux. Sagittal reformatted CBCT demonstrating the presence of this anatomical variant (arrow)

Another useful application of CBCT is the imaging of various anatomic variants, bony coalitions, osseous defects simulating cartilage lesions on other imaging modalities (Fig. 13) or accessory or bifid bones (Fig.14).

## Interventional radiology and future developments

Some modern flat panel detector C-arm units combine fluoroscopy with CBCT imaging offering guidance for interventional radiology procedures [28]. Spinal interventions including nucleoplasty, vertebroplasty or bone biopsies are among the most frequently performed percutaneous interventional musculoskeletal procedures. More and more ablation or palliative procedures are performed under imaging guidance, including CBCT [29]. When precise biopsy needle positioning is difficult to achieve, CBCT application can help in choosing the best approach, precise tracking and detection of errors in the operating room. The reduction of cumulative dose to patient and staff can be achieved by reduction of fluoroscopy time due to the usage of CBCT guidance [28, 30]. Fusion with MRI for precise lesion targeting has been reported as well [31]. The overall duration of those procedures under MDCT and CBCT guidance are similar with less dose applied to both the patient and performing physician for CBCT [32]. Especially in young patients, lower radiation dose is of utmost importance. Other systems on the market are designed for weight-bearing extremities examinations, allowing evaluation of joint stability [20]. Given the specific design (the size of the gantry, presence of guide lights for needle position) for each application, every business case for purchasing CBCT equipment should be tailored to the special needs in each department.

## Conclusions

CBCT is a promising method that may be very useful for evaluation of trauma of small joints and bones, particularly when CR is negative or doubtful despite high clinical suspicion for fractures. In combination with arthrography, CBCT offers high anatomical detail of articular cartilage, which may be an advantage to routine MRI in evaluation of osteochondral lesions in joints with thin cartilage such as the ankle joint. Furthermore, the technique may serve as an alternative method to MRI for a variety of musculoskeletal diseases in patients with claustrophobia or other contraindications of MRI. In all these scenarios, an improved diagnosis may result in a more timely and appropriate treatment regime. Although MDCT equipment remains the preferred CT technology for multifunctional purposes in most imaging departments, due to its low cost in purchase and maintenance, CBCT may be a useful tool in private practices with a high turn-over of musculoskeletal procedures or as an additional imaging tool to MDCT in large hospitals. Awareness of the advantages and disadvantages of the technique is a prerequisite for its dedicated use.

## Abbreviations

CBCT: Cone Beam Computed Tomography
CBCT-A: Cone Beam Computed Arthrography
CR: Conventional Radiography
FOV: Field of View
HU: Hounsfield Units
kVp: KiloVoltage power
mAs: Milliamperage seconds
MDCT: Multidetector Computed Tomography
MRI: Magnetic Resonance Imaging
